# MMPP promotes adipogenesis and glucose uptake *via* binding to the PPARγ ligand binding domain in 3T3-L1 MBX cells

**DOI:** 10.3389/fphar.2022.994584

**Published:** 2022-10-21

**Authors:** Na-Yeon Kim, Chae-Min Lim, Hyo-Min Park, Jinju Kim, Thu-Huyen Pham, Young Yang, Hee Pom Lee, Jin Tae Hong, Do-Young Yoon

**Affiliations:** ^1^ Department of Bioscience and Biotechnology, Konkuk University, Seoul, Korea; ^2^ Department of Biological Science, Sookmyung Women’s University, Seoul, Korea; ^3^ College of Pharmacy & Medical Research Center, Chungbuk National University, Cheongju, Korea

**Keywords:** MMPP, PPARγ agonist, type 2 diabetes treatment, adipogenesis, glucose uptake

## Abstract

Peroxisome proliferator-activated receptor-gamma (PPARγ) is a transcription factor involved in adipogenesis, and its transcriptional activity depends on its ligands. Thiazolidinediones (TZDs), well-known PPARγ agonists, are drugs that improve insulin resistance in type 2 diabetes. However, TZDs are associated with severe adverse effects. As current therapies are not well designed, novel PPARγ agonists have been investigated in adipocytes. (E)-2-methoxy-4-(3-(4-methoxyphenyl) prop-1-en-1-yl) phenol (MMPP) is known to have anti-arthritic, anti-inflammatory, and anti-cancer effects. In this study, we demonstrated the adipogenic effects of MMPP on the regulation of PPARγ transcriptional activity during adipocyte differentiation *in vitro*. MMPP treatment increased PPARγ transcriptional activity, and molecular docking studies revealed that MMPP binds directly to the PPARγ ligand binding domain. MMPP and rosiglitazone showed similar binding affinities to the PPARγ. MMPP significantly promoted lipid accumulation in adipocyte cells and increased the expression of C/EBPβ and the levels of p-AKT, p-GSK3, and p-AMPKα at an early stage. MMPP enhanced the expression of adipogenic markers such as PPARγ, C/EBPα, FAS, ACC, GLUT4, FABP4 and adiponectin in the late stage. MMPP also improved insulin sensitivity by increasing glucose uptake. Thus, MMPP, as a PPARγ agonist, may be a potential drug for type 2 diabetes and metabolic disorders, which may help increase adipogenesis and insulin sensitivity.

## 1 Introduction

A recent report by the World Health Organization (WHO) estimated that approximately 13% of the world’s adult population was obese in 2016 (World Health Organization, 2021). Obesity is a global health concern. Obesity is linked to diseases such as cardiovascular disease, type 2 diabetes (T2D), hypertension, certain cancers, and stroke (Poirier et al., 2006; Haley and Lawrence, 2016). Among these, the incidence of T2D has significantly increased. T2D is caused by insulin resistance and is defined as the inability of insulin to increase glucose uptake and utilization (Lebovitz, 2001; Wu et al., 2014). Adipose tissue must take up glucose and regulate adipogenesis to decrease blood glucose levels.

Peroxisome proliferator-activated receptor-gamma (PPARγ) is a key regulator of adipogenesis, lipid metabolism, inflammation, and metabolic homeostasis (Lehmann et al., 1995; Wang et al., 2014; Wang et al., 2016). PPARγ agonists such as thiazolidinediones (TZDs) have been used in type 2 diabetes treatment to increase insulin sensitivity (Henke et al., 1998; Farmer, 2005). However, TZDs have severe side effects such as weight gain, fluid retention, bone loss, congestive heart failure, and a possible increased risk of myocardial infarction and bladder cancer (Wang et al., 2016). As current therapies are not well designed, novel PPARγ agonists that regulate adipogenesis must be investigated to control type 2 diabetes and other obesity-related health problems.

(E)-2-methoxy-4-[3-(4-methoxyphenyl) prop-1-en-1-yl] phenol (MMPP) is a synthetic (E)-2,4-bis(p-hydroxyphenyl)-2-butenal (BHPB) analog that exerts anti-inflammatory and anti-arthritic effects by inhibiting the activation of STAT3 (Son et al., 2016). Recently, it has been reported that MMPP exerted anti-tumor activity in a patient-derived non-small cell lung cancer xenograft model (Son et al., 2017). Inflammation is also closely associated with adipogenesis (Cox et al., 2015). However, the functional effects of MMPP on adipose tissue have not been elucidated yet. If MMPP can act as a PPARγ agonist, it can bind to and activate PPARγ; thus, it may be a candidate for modulating metabolic disorders. Therefore, in this study, we aimed to evaluate the effects of MMPP on lipid accumulation and its PPARγ promoter activity to explore the underlying molecular mechanisms in adipose tissue. These results will help understand the mechanisms of metabolic disorders such as obesity, diabetes, and hyperlipidemia.

## 2 Materials and methods

### 2.1 Reagents

MMPP, kindly donated by Dr. Hong JT (Chungbuk National University, Cheongju, Korea), was produced as previously described (Son et al., 2016). 3-Isobutyl-1-methylxanthine (IBMX), dexamethasone (DEX), insulin, rosiglitazone, and Oil Red O were purchased from Sigma-Aldrich (St. Louis, MO, United States).

### 2.2 Cell culture and differentiation

3T3-L1 MBX cells were obtained from the American Type Culture Collection (Manassas, VA, United States , #CL-173) and cultured in Dulbecco’s modified Eagle’s medium (Welgene Incorporation, Daegu, Korea) containing 10% heat-inactivated fetal bovine serum (Hyclone Laboratories, Logan, UT, United States), penicillin (100 U/mL), and streptomycin (100 U/mL), at 37°C in a humidified atmosphere containing 5% CO_2_ and subjected to a maximum of 10 passages. 3T3-L1 MBX preadipocytes were seeded in 6-well plates (3 × 10^4^ cells/well) and incubated for 4 days until confluence. After 2 days of confluence (day 0), the cells were differentiated in DMEM supplemented with 10% FBS, 0.5 mM IBMX, 1 μM DEX, and 10 μg/ml insulin for 2 days. On day 2, the medium was changed to DMEM containing FBS and 10 μg/ml insulin, in the presence or absence of MMPP at the indicated concentrations, and then the cells were cultured for another 2 days. On day 4, the cells were maintained in DMEM with FBS, and the medium was changed every 2 days until day 8. MMPP was added each time the culture medium was changed. In the same way, rosiglitazone was treated as a positive control.

### 2.3 Oil Red O staining

Fully differentiated cells were washed with phosphate-buffered saline (PBS) and fixed with 4% formaldehyde for 1 h. The cells were then rinsed with 60% isopropyl alcohol for 20 min and completely dried. Next, the cells were stained with Oil Red O solution (0.2 mg/ml Oil Red O in isopropanol) for at least 30 min at RT. The stained lipid droplets were observed under a light microscope (Nikon, Tokyo, Japan). The stained oil droplets were dissolved in 100% isopropanol, and the absorbance was measured at 492 nm using a spectrophotometer (Al Hasan et al., 2021).

### 2.4 2-deoxyglucose uptake assay

The Glucose Uptake-Glo^™^ Assay (Promega, Madison, WI, United States) was performed after incubation with MMPP or rosiglitazone in mature adipocytes (Day 8). Immediately before starting the experiment, the culture medium was discarded, and the cells were washed with PBS to remove the remaining glucose. Next, cells were treated with 1 mM 2-deoxyglucose (2-DG) for 10 min. The cells were then subjected to further processing according to the manufacturer’s protocol. After a brief incubation period, an acid detergent solution (stop buffer) was added. Next, a neutralization buffer was added to neutralize the acid, followed by a detection reagent.

### 2.5 Cell viability assay

Cell viability was measured using 3-(4,5-dimethylthiazol-2-yl)-5-(3-carboxy methoxy phenyl)-2-(4-sulfophenyl)-2H-tetrazolium (MTS) assay. 3T3-L1 MBX cells were seeded in 100 μL complete culture medium in 96-well plates and treated with MMPP for 48 h. The effect of MMPP on cell viability was measured using the CellTiter 96 Aqueous One Solution Assay (Promega, Madison, WI, United States ) containing MTS and phenazine methosulfate, an electron-coupling reagent. Briefly, a 100-μL aliquot of the aqueous reagent solution was added to each well, and the cells were incubated for 1 h. Absorbance was measured at 492 nm using a microplate reader (Apollo LB 9110; Berthold Technologies GmbH, Bad Wildbad, Germany). The percentage of viable cells was estimated relative to that of the untreated controls.

### 2.6 RNA isolation and reverse-transcription polymerase chain reaction

Total RNA was isolated using the easy-BLUE Total RNA Extraction Kit (iNtRON, Seoul, Korea). First-strand cDNA was synthesized with oligo (dT) primers using M-MulV reverse transcriptase (New England Biolabs, Ipswich, MA, United States ). The synthesized first-strand cDNA was used for the PCR amplification of specific genes. The primer sequences used to amplify the target genes were as follows: *Pparg* (sense, 5′-GTA​CTG​TCG​GTT​TCA​GAA​GTG​CC-3′; antisense, 5′-ATC​TCC​GCC​AAC​AGC​TTC​TCC​T-3′), *C/ebpa* (sense, 5′-TCG​GTG​GAC​AAG​AAC​AGC​AA-3′; antisense, 5′-TTG​TCA​CTG​GTC​AGC​TCC​AG-3′), *Fas* (sense, 5′-CAC AGTGCTCAAAGG ACATGCC-3′; antisense, 5′-CAC​CAG​GTG​TAG​TGC​CTT​CCT​C-3′), *Acc* (sense, 5′-GTT​CTG​TTG​GAC​AAC​GCC​TTC​AC-3′; antisense, 5′-GGA​GTC​ACA​GAA​GCA​GCC​CAT​T-3′), *Glut4* (sense, 5′-GGT​GTG​GTC​AAT​ACG​GTC​TTC​AC-3′; antisense, 5′-AGC​AGA​GCC​ACG​GTC​ATC​AAG​A-3′), *Fabp4* (sense, 5′-TGA​AAT​CAC​CGC​AGA​CGA​CAG​G-3′; antisense, 5′-GCT​TGT​CAC​CAT​CTC​GTT​TTC​TC-3′), *Adiponectin* (sense, 5′-AGA​TGG​CAC​TCC​TGG​AGA​GAA​G-3′; antisense, 5′-ACA​TAA​GCG​GCT​TCT​CCA GGCT-3′), *Il6* (sense, 5′-TAC​CAC​TTC​ACA​AGT​CGG​AGG​C-3′; antisense, 5′-CTG​CAA​GTG​CAT​CAT​CGT​TGT​TC-3′), and *Gapdh* (sense, 5′-CAT​CAC​TGC​CAC​CCA​GAA​GAC​TG-3′; antisense, 5′-ATG​CCA​GTG​AGC​TTC​CCG​TTC​AG-3′). *Gapdh* was used as the RNA-loading control. PCR products were separated by electrophoresis on 2% agarose gels and detected by ethidium bromide staining.

### 2.7 Quantitative real-time PCR

Real-time PCR was performed using a relative quantification procedure on a Thermal Cycler Dice Real Time System with TB Green Premix Ex Taq (Takara Bio, Otsu, Japan) for amplification detection. The primer of sequences used to amplify the target genes were as follows: *Gapdh* (sense, 5′-CAT​CAC​TGC​CAC​CCA​GAA​GAC​TG-3′; antisense, 5′-ATG​CCA​GTG​AGC​TTC​CCG​TTC​AG -3′) and *C/ebpb* (sense, 5′-CAA​CCT​GGA​GAC​GGC​ACA​AG-3′; antisense, 5′- GCT​TGA​ACA​AGT​TCC​GCA​GGG​T-3′). The results were analyzed using the TaKaRa Dice Real-Time System Single (Takara Bio, Otsu, Japan). All the expression values of the target genes were normalized to GAPDH expression as a housekeeping control. Quantitative gene expression values were calculated using the ΔΔCT method with data from independent triplicate experiments.

### 2.8 Immunoblot analysis

3T3-L1 MBX cells (3 × 10^4^ cells/well) were seeded in 60 mm cell culture dishes and differentiated for 8 days. The cells were lysed in a buffer containing 50 mM Tris (pH 7.4), 150 mM NaCl, 1% NP40, 0.1% sodium dodecyl sulfate (SDS), 0.25% sodium deoxycholate, 1 mM orthovanadate, aprotinin (10 μg/ml), and 0.4 mM phenylmethylsulfonyl fluoride at 4°C for 30 min. Equal amounts of total cellular protein (50 μg) isolated from the harvested cells were separated by SDS-PAGE and transferred onto a PVDF membrane (Choi et al., 2022). Specific proteins were detected using antibodies against PPARγ, CCAAT/enhancer-binding protein (C/EBP) α, phosphorylated protein kinase B (PKB/AKT), glyceraldehyde 3-phosphate dehydrogenase (GAPDH), C/EBPβ, β-actin and phosphorylated glycogen synthase kinase three α/β (GSK3 α/β) (Santa Cruz Biotechnology, Santa Cruz, CA, United States ). Antibodies against fatty acid synthase (FAS) and acetyl-CoA carboxylase (ACC) and glucose transporter type 4 (GLUT4) were purchased from Cell Signaling Technology (Danvers, MA, United States).

### 2.9 Molecular docking studies

A docking study of MMPP with the PPARγ ligand binding domain (LBD) was performed using AutoDock VINA v1.2.0 (Trott and Olson, 2010). The crystal structures of PPARγ LBD (PDB code: 2PRG) were used in the docking experiments (Kumar et al., 2018; Kores et al., 2021). The grid box was centered on the PPARγ LBD and its size was adjusted to include the whole protein. Molecular graphics for the best binding model were generated using Biovia Discovery Studio Visualizer v21.1.0 and UCSF Chimera v1.16 (Pettersen et al., 2004).

### 2.10 Transcriptional activity assay

The effect of MMPP on PPARγ transcriptional activity was investigated by conducting the luciferase assay on the human embryonic kidney (HEK) 293T cells. The cells were cultured in DMEM containing 10% FBS and seeded in 24-well plates (1.0 × 10^5^ cells/well). On the next day, the cells were transiently transfected with plasmids expressing PPARγ (0.2 μg/well), (PPAR response element × 3)-thymidine kinase-luciferase reporter construct (0.2 μg/well), and *Renilla* luciferase control vector pRL (0.1 μg/well) using JetOPTIMUS reagent (Polyplus, Iillkirch, France) for 24 h. The plasmids expressing PPARγ and the reporter construct were prepared as previously described (Kim et al., 2012; Song et al., 2016). After treatment with MMPP (15 μg/ml) or rosiglitazone (1 μM) for 24 h, the cells were harvested and assayed using a dual-luciferase reporter gene assay kit (Promega, Madison, WI, United States ). The assay results were reported in relative luciferase activity units and calculated as the ratio of the expression of firefly luciferase to *Renilla* luciferase.

### 2.11 Statistical analysis

Statistical analysis was conducted using one-way analysis of variance with Tukey’s honestly significant difference tests. Differences were considered statistically significant at *p* < 0.05. Results were obtained from at least three separate experiments and expressed as the mean ± SD.

## 3 Results

### 3.1 Docking studies revealed that MMPP binds to the PPARγ LBD

**FIGURE 1:**
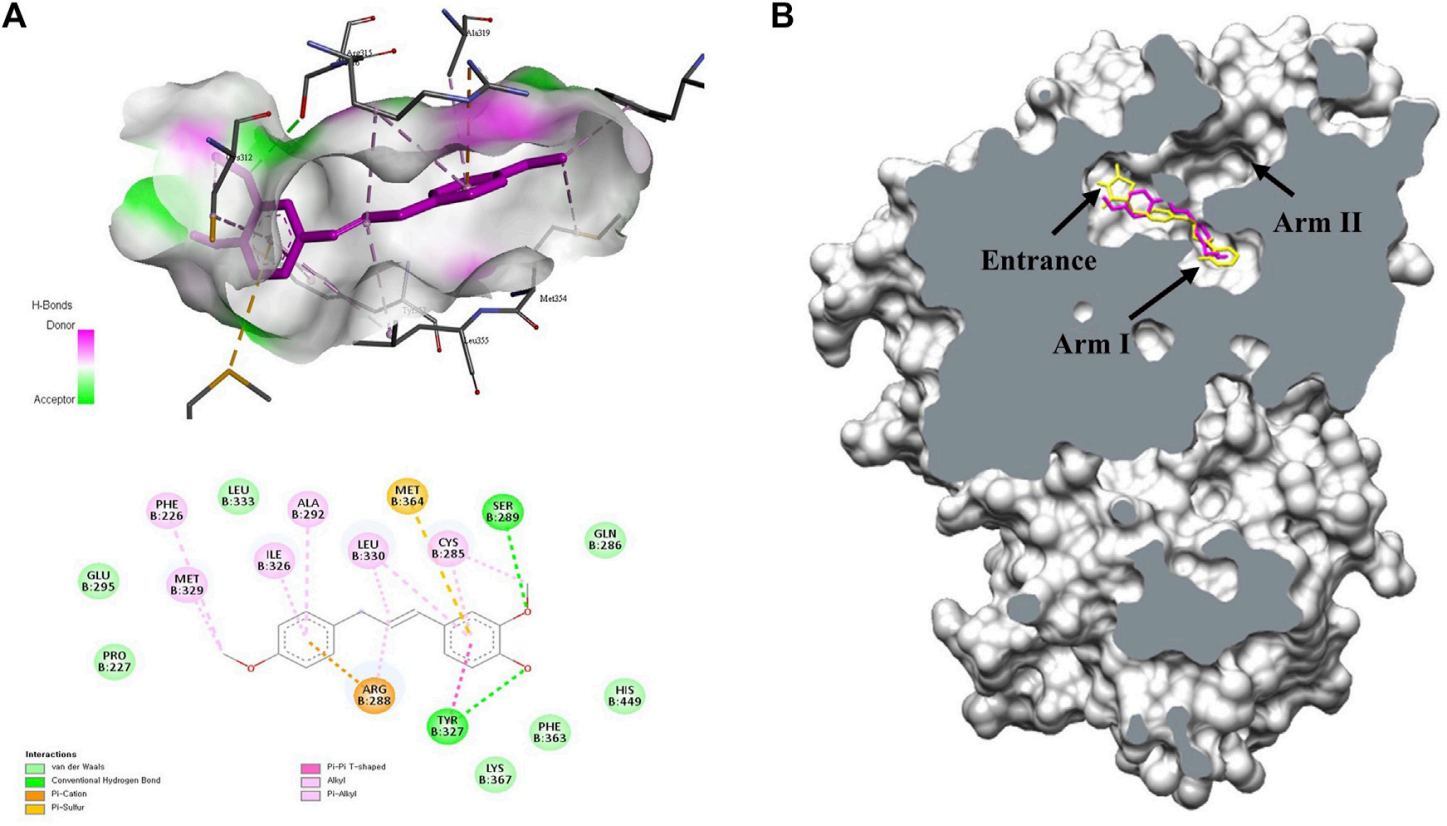
Molecular docking studies on the interactions between MMPP or rosiglitazone and the PPARγ ligand binding domain (LBD). **(A)** 3D and 2D diagram of docking complex of the PPARγ LBD (PBD code: 2PRG) with MMPP (CID:122517441). The most favorable docking result was described by the Biovia Discovery Studio visualizer. **(B)** The capped surface of the docking complex of the PPARγ LBD with rosiglitazone (CID:77999; yellow) and MMPP (pink) performed using UCSF Chimera. They docked at Arm one of the binding pocket in PPARγ LBD.

To understand the mechanism of PPARγ activation by MMPP, a docking study was performed between MMPP and the crystal structure of the PPARγ LBD. MMPP formed hydrogen bonds with amino acid residues Ser289 and Tyr327 of the PPARγ LBD, with bond lengths of 2.96 Å and 2.93 Å, respectively. MMPP participated in hydrophobic interactions with the amino acid residues Phe226, Cys285, Gln286, Glu295, Arg299, Ile326, Met329, Leu330, Phe363, Met364, and His449 (Figure 1A). To compare MMPP to rosiglitazone, a PPARγ agonist, a docking study was performed between rosiglitazone and PPARγ LBD under the same conditions. The MMPP-binding pose was similar to that of rosiglitazone. Both were bound to the binding pockets of PPARγ LBD (Figure 1B) with high binding affinities of −8.0 and −8.9 kcal/mol, respectively. These results suggested that MMPP could directly bind to PPARγ, and its binding pose was similar to that of rosiglitazone.

### 3.2 MMPP upregulated the transcriptional activity of PPARγ

**FIGURE 2:**
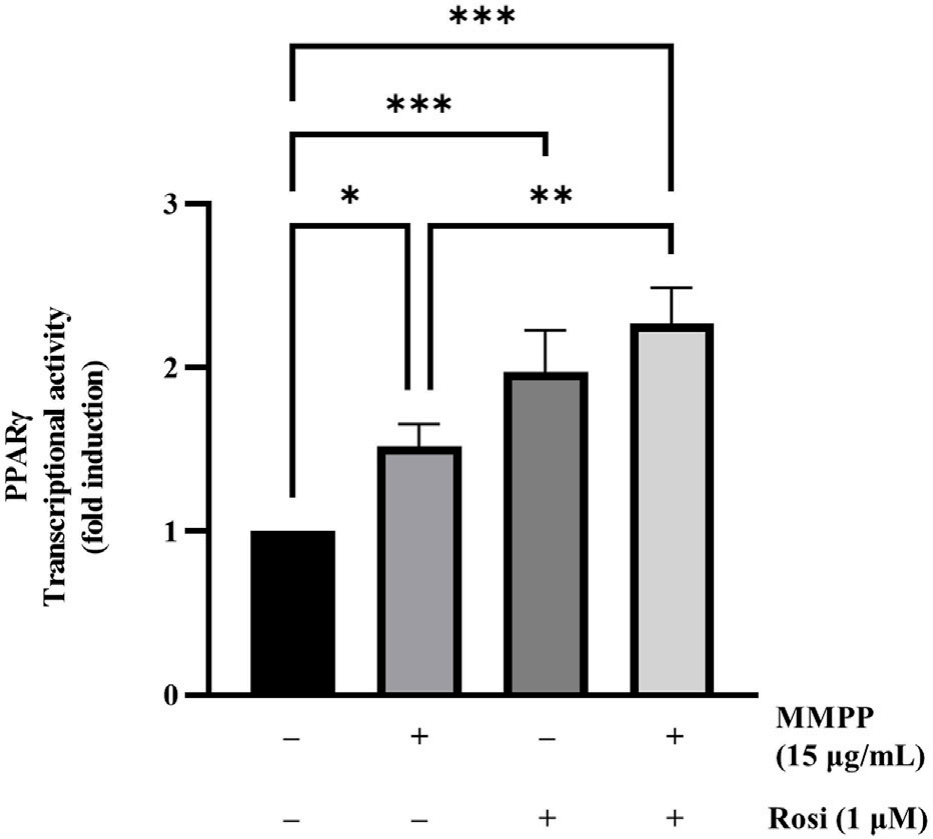
The effect of MMPP on PPARγ transcriptional activity. PPARγ transcriptional activity was assessed using a luciferase assay. HEK 293T cells were seeded in a 24-well plate (1.0 × 10^5^ cells/well) and transfected with plasmids expressing PPARγ, (PPAR response element × 3)-thymidine kinase-luciferase reporter constructs, and the *Renilla* luciferase control vector pRL for 24 h. Then, the cells were treated with MMPP or rosiglitazone for another 24 h. Finally, the cells were harvested, and a luciferase assay was performed to examine the transcriptional activity of PPARγ. Data are from three independent experiments and reported as mean ± SD (*n* = 3). *p < 0.05, **p < 0.01, ***p < 0.001.

We performed a transcriptional activity assay to verify the effects of MMPP on PPARγ expression. HEK 293T cells were transfected with PPARγ and (PPRE × 3)-tk-luciferase expression vectors, followed by treatment with MMPP. As shown in Figure 2, MMPP and rosiglitazone, both increased the transcriptional activity of PPARγ, as measured by luciferase assay. PPARγ activity was further enhanced when MMPP and rosiglitazone were co-administered. These results show that MMPP can be a potential PPARγ agonist.

### 3.3 MMPP did not affect the viability of 3T3-L1 MBX cells

**FIGURE 3:**
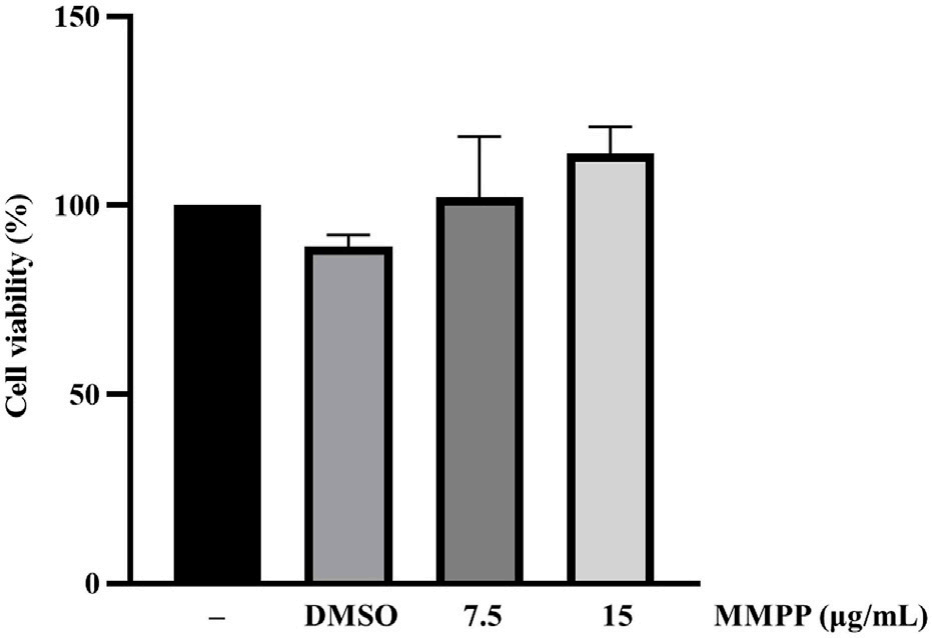
Cytotoxic effects of MMPP on 3T3-L1 MBX cells. 3T3-L1 MBX cells were treated with DMSO (0.1%) and MMPP (7.5 or 15 μg/ml) for 48 h. Data are from three independent experiments and reported as mean ± SD (*n* = 3).

The cytotoxicity of MMPP was assessed in 3T3-L1 MBX cells using the MTS assay. In this study, we evaluated the functional effect of MMPP on adipogenesis by exposing the cells to non-cytotoxic concentrations. MMPP and rosiglitazone were dissolved in DMSO. DMSO (0.1%) has no cytotoxic effect on the cells. All experiments were performed using less than 0.1% DMSO. DMSO was used as the vehicle control in all experiments. MMPP showed no cytotoxic effects at concentrations up to 15 μg/ml (Figure 3).

### 3.4 MMPP promoted lipid accumulation and glucose uptake in 3T3-L1 MBX cells

**FIGURE 4:**
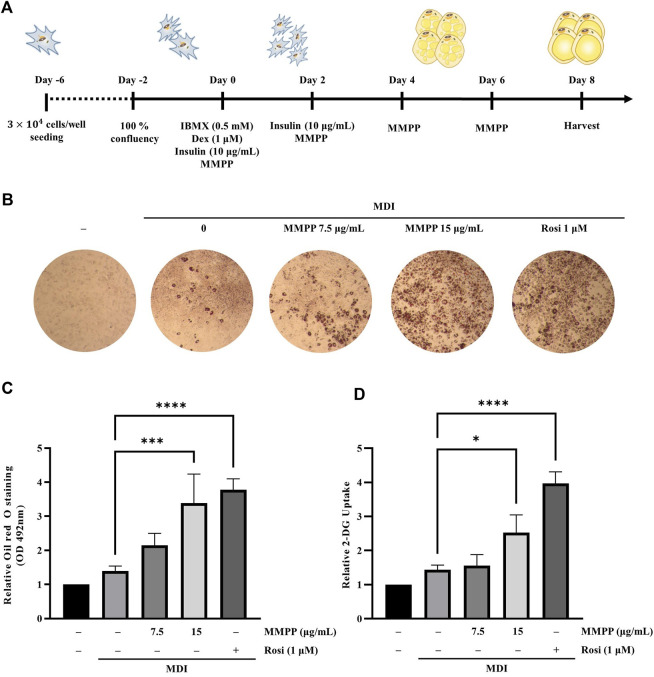
The effect of MMPP on lipid accumulation in 3T3-L1 MBX cells. **(A)** Diagram of the differentiation schedule. 3T3-L1 MBX cells were differentiated using MDI (IBMX, dexamethasone, and insulin), and then treated with MMPP or rosiglitazone. **(B)** Photograph of the plate after Oil Red O staining. Briefly, after 8 days of differentiation, the cells were fixed with 10% formaldehyde and stained with Oil Red O solution. The stained cells were then observed under a microscope. Representative images from four independent experiments are shown. **(C)** Accumulated lipids were stained with Oil Red O, eluted, and quantitated using spectrophotometric analyses at 492 nm. Data are from four independent experiments and reported as the mean ± SD (*n* = 4). ***p < 0.001, ****p < 0.0001 *versus* MDI group. **(D)** The effects of MMPP on the glucose uptake in mature adipocytes. Data are from three independent experiments and reported as mean ± SD (*n* = 3). *p < 0.05, ****p < 0.0001 *versus* MDI group.

To examine the effects of MMPP on lipid accumulation, 3T3-L1 MBX cells were differentiated with MMPP (7.5 or 15 μg/ml) or rosiglitazone (1 μM, as a positive control) (Figure 4A). Differentiated adipocytes were stained with Oil Red O to measure their lipid content. As shown in Figures 4B,C, more lipid droplets were detected in MMPP treated 3T3-L1 MBX cells in a dose-dependent manner. Rosiglitazone, a well-known PPARγ agonist, promoted lipid accumulation in 3T3-L1 MBX cells. MMPP-induced glucose uptake was evaluated by 2-DG uptake assay. 2-DG uptake assay demonstrated that MMPP also increased glucose uptake in 3T3-L1 MBX cells (Figure 4D). These results indicate that MMPP as well as rosiglitazone stimulated adipogenesis and glucose uptake in 3T3-L1 MBX cells.

### 3.5 MMPP enhanced adipogenesis by increasing the expression of adipogenesis-related genes and proteins in 3T3-L1 MBX cells

**FIGURE 5:**
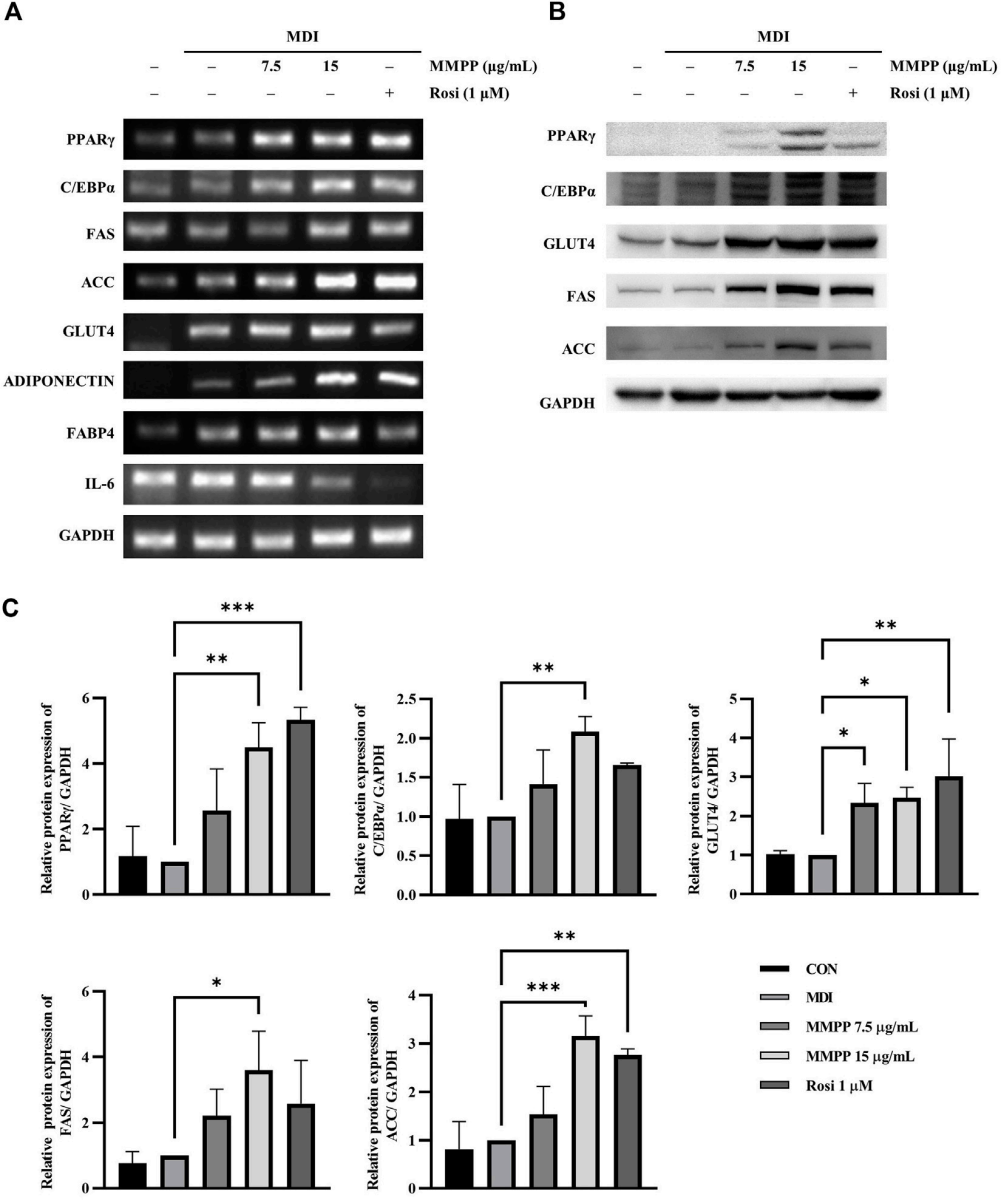
The effects of MMPP on the expression of adipogenesis-related factors and glucose uptake. **(A)** 3T3-L1 MBX cells were differentiated using MDI (IBMX, dexamethasone, and insulin) and treated with MMPP or rosiglitazone. After 8 days, the differentiated cells were harvested and analyzed using RT-PCR to detect the mRNA expression levels of *Pparg*, *C/ebpa*, *Fas*, *Acc*, *Glut4*, *Adiponectin*, *Fabp4 and Il6*. *Gapdh* was used as the loading control. Similar results obtained in three independent experiments (*n* = 3). **(B)** Protein expression levels of PPARγ, C/EBPα, GLUT4, FAS, ACC, and GAPDH. Fully differentiated 3T3-L1 MBX cells treated with MMPP or rosiglitazone were harvested, and immunoblotting was performed. Similar results obtained in three independent experiments (*n* = 3). **(C)** Bar graphs show densitometric quantification of protein expression. Data are from three independent experiments and reported as the mean ± SD (*n* = 3). *p < 0.05, **p < 0.01, ***p < 0.001 *versus* MDI group.

To investigate the effect of MMPP on adipognesis, the expression levels of genes and proteins involved in adipogenesis were investigated in fully differentiated 3T3-L1 MBX cells treated with MMPP or rosiglitazone. RT-PCR analyses showed that MMPP promoted the expression of transcription factors (*Ppar*g and *C/ebpa*) and adipogenic markers (*Fas, Acc, Adiponectin, Fabp4*, and *Glut4*) (Figure 5A). However, *Il6* mRNA levels were reduced by MMPP treatment. Immunoblot analyses revealed that the protein expression levels of adipogenic markers, such as PPARγ, C/EBPα, GLUT4, FAS, and ACC, were increased (Figures 5B,C). Taken together, these results indicate that MMPP enhanced adipogenesis by increasing the expression of adipocyte differentiation-related factors.

### 3.6 MMPP upregulated C/EBPβ expression as well as phosphorylation levels of AKT, GSK3 and AMPKα during early stages of adipogenesis

**FIGURE 6:**
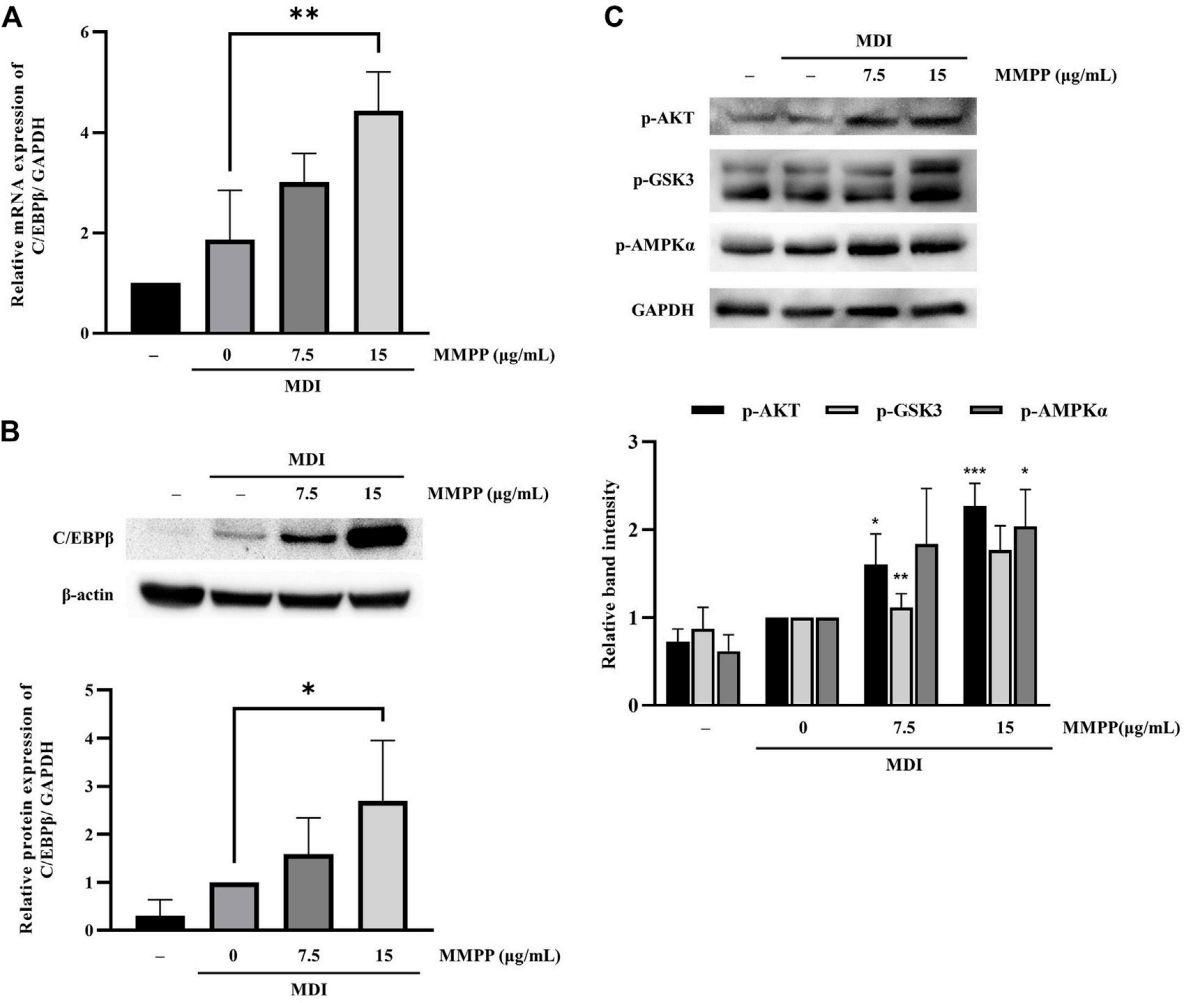
The effect of MMPP on early adipogenesis. **(A)** The mRNA expression level of *C/ebpb* on day 2 of differentiation was analyzed using real-time PCR. Data are from three independent experiments and reported as the mean ± SD (*n* = 3). **p < 0.01 *versus* MDI group. **(B)** The protein expression levels of C/EBPβ on day 2 were assessed by immunoblotting. Bar graph shows the signal intensity of protein bands in arbitrary units after normalization with the signal intensity of β-actin internal control for each sample. Data are from four independent experiments and reported as the mean ± SD (*n* = 4). *p < 0.05 *versus* MDI group. **(C)** 3T3-L1 MBX cells were stimulated with MDI and treated with MMPP for 1 h p-AKT, p-GSK and p-AMPKα were evaluated by immunoblotting. Bar graph shows the signal intensity of protein bands in arbitrary units after normalization with the signal intensity of GAPDH for each sample. Data are from three independent experiments and reported as the mean ± SD (*n* = 3). *p < 0.05, **p < 0.01, ***p < 0.001 *versus* MDI group.

**FIGURE 7:**
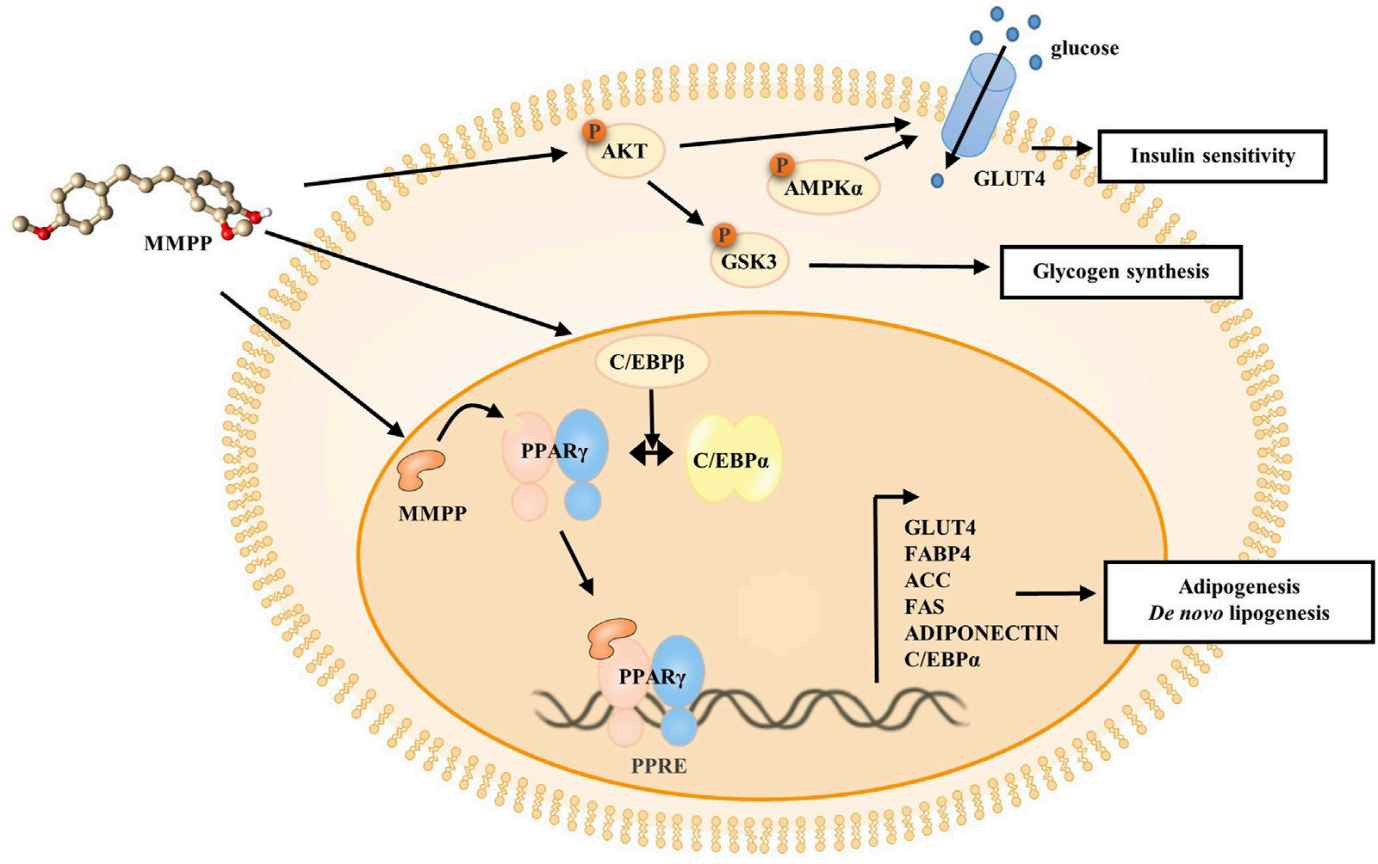
Schematic diagram of the effect of MMPP on the 3T3-L1 MBX cells. MMPP increases the expression of C/EBPβ, as well as levels of p-AKT, p-GSK3, and p-AMPKα at an early stage. MMPP enhances GLUT4 expression and glucose uptake, and MMPP-induced AKT/AMPK activation can regulate GLUT4 translocation. MMPP-induced phosphorylation and inactivation of GSK3 can lead to enhanced glycogen synthesis. Thus, MMPP can increase insulin sensitivity. MMPP binds to PPARγ and promotes transcriptional activity of PPARγ. It enhances the expression of adipogenic markers (C/EBPα, FAS, ACC, GLUT4, ADIPONECTIN, and FABP4) in the late stage, resulting in adipogenesis and *de novo* lipogenesis.

To investigate the effects of MMPP on early stages of differentiation, we examined the expression of factors related to early adipogenesis. 3T3-L1 MBX cells were differentiated using MDI, treated with MMPP (7.5 or 15 μg/ml) for 48 h, and the expression of early adipogenesis-related factors was analyzed using real-time PCR and immunoblotting. As shown in Figures 6A,B, both *C/ebpb* gene and C/EBPβ protein expression levels were increased by MMPP. These results suggest that MMPP can promote adipogenesis by modulating the expression of early adipogenesis-related factors. Many signaling pathways were involved in early adipogenesis such as phosphoinositide 3-kinase/protein kinase B (PI3K/AKT) and AMP-activated protein kinase (AMPK) pathways (Song et al., 2016; Chang and Kim, 2019; Gunasinghe et al., 2019; Li et al., 2020). To investigate how MMPP modulates early adipogenesis, phosphorylation levels of AKT, GSK3, and AMPKα were evaluated by immunoblotting. 3T3-L1 MBX cells were treated with MMPP and MDI for 1 h. MMPP enhanced the phosphorylation of AKT, GSK3 and AMPKα (Figure 6C). These results indicated that MMPP phosphorylated AKT, GSK3 and AMPKα, followed by upregulation of C/EBPβ expression, resulting in the activation of signaling pathways involved in adipogenesis (Figure 7).

## 4 Discussion

The International Diabetes Federation (IDF) has reported that the number of adults living with diabetes was estimated to be 425 million in 2017. The IDF predicted that 578 million people will have diabetes by 2030, without sufficient action. Furthermore, >90% of individuals with diabetes are diagnosed with type 2 diabetes (T2D) (Saeedi et al., 2019). T2D is a global problem that must be resolved. The body’s system maintains glucose levels through the pair of opposing hormones, insulin and glucagon (Unger and Orci, 2010). However, type 2 diabetics have low insulin sensitivity, impaired glucose tolerance, and decreased insulin-stimulated glucose uptake; therefore, glucose level becomes too high (Lebovitz, 2001; Sanches et al., 2021). PPARγ is a key regulator of adipogenic differentiation, and its role has been well established. PPARγ regulates insulin sensitivity, lipid storage, *de novo* lipogenesis, and adipocyte differentiation, and it influences insulin sensitivity in adipose tissue, liver, and skeletal muscle (Rosen et al., 1999; Cariello et al., 2021). Thus, PPARγ agonists can be used as drugs against metabolic disorders, including T2D, by improving insulin sensitivity and reducing plasma glucose concentration (Huang et al., 2012).

In this study, MMPP was showed to be a PPARγ agonist. Docking studies revealed that MMPP directly binds to PPARγ LBD (Figure 1). The PPARγ LBD consists of 13 helices and a four-stranded β-sheet. It has a Y-shaped ligand binding cavity composed of two pockets, Arm I, Arm II, and the entrance, as shown in Figure 1B (Fyffe et al., 2006). Arm I is a hydrophilic pocket that includes a residue of the activation function 2 (AF2) helix (Zoete et al., 2007). This pocket forms hydrogen bonds with the acidic head group of rosiglitazone. This interaction stabilizes the structure of the PPARγ LBD and leads to the recruitment of coactivators, resulting in the remodeling of chromatin and recruitment of transcriptional machinery (Helsen and Claessens, 2014). Docking studies indicated that the -OH functional groups of MMPP formed hydrogen bonds with the amino acid residues Ser289 and Tyr237 of Arm I. Thus, MMPP can directly bind to PPARγ, resulting in the stabilization of AF2, recruitment of PPARγ coactivator, and stimulation of PPARγ transcriptional activity. Luciferase assay using PPARγ and (PPRE × 3)-tk-luciferase expression vectors revealed that MMPP increased PPARγ transcriptional activity (Figure 2). These data support the hypothesis that MMPP is a PPARγ agonist.

We investigated the effects of MMPP on the adipogenesis of 3T3-L1 preadipocytes. MMPP promoted the expression of adipogenic markers during 3T3-L1 MBX differentiation. MMPP enhanced the adipogenic marker expression of *Fas, Acc, Fabp4, Adiponectin, and Glut4* by increasing the expression of transcription factors such as Pparg and C/ebpa, resulting in the promotion of lipid accumulation in 3T3-L1 MBX cells. MMPP treatment reduced Il6 mRNA levels compared to those in the MDI-treated group (Figure 5A). PPARγ and C/EBPα are the key adipogenic transcription factors. Extensive crosstalk between PPARγ and C/EBPα induces transcriptional activation of adipogenic genes such as *Acc*, *Fas*, *Adiponectin*, *Glut4* and *Fabp4* (Siersbaek et al., 2012). ACC initiates *de novo* lipogenesis by catalyzing the carboxylation of acetyl-CoA to malonyl-CoA. FAS then synthesizes fatty acids from acetyl-CoA and malonyl-CoA (Yuan et al., 2015; Sanders and Griffin, 2016). In the MMPP-treated group, ACC and FAS levels were increased compared to those in MDI-treated group (Figures 5B,C). Thus, MMPP activated *de novo* lipogenesis. ADIPONECTIN is an adipokine secreted by adipose tissue. It can activate the insulin pathway, resulting in increased lipogenesis, glucose uptake, glycogen synthesis, and reduced lipolysis and gluconeogenesis (Yadav et al., 2013; Achari and Jain, 2017). The decreased IL-6 expression in the adipocytes can induce adiponectin secretion and GLUT4 expression (Rotter et al., 2003). GLUT4 increases insulin sensitivity, which lowers blood glucose levels by upregulating glucose levels (Govers, 2014). IL-6 downregulates insulin signaling and causes insulin resistance in adipocytes. The increase in ADIPONECTIN and GLUT4 expression and the decrease in IL-6 expression suggest that MMPP treatment recovers insulin sensitivity. FABP4 is involved in lipid trafficking. FABP4 acts as a lipid chaperone to take up lipids and fatty acids under pathophysiological conditions and can be a therapeutic target for metabolic disorders (Furuhashi et al., 2014; Furuhashi, 2019). MMPP was found to upregulate adipogenesis-related genes and proteins, suggesting that MMPP exerts insulin-sensitizing effects, similar to a PPARγ agonist (Figure 7).

Next, we investigated the effect of MMPP on early adipogenesis. C/EBPβ and C/EBPδ have been reported to contribute to the mitotic clonal expansion of 3T3-L1 MBX cells (Hishida et al., 2009). C/EBPβ and C/EBPδ are activated to promote PPARγ and C/EBPα, which are crucial transcription factors in late adipogenesis (Hassan et al., 2012). In the present study, we found that MMPP increased expression of C/EBPβ (Figure 6B).

Glucose homeostasis must be maintained to overcome T2D. MMPP phosphorylates AKT, GSK3 and AMPKα (Figure 6C) which are protein kinases associated with glucose homeostasis (Schultze et al., 2012). When Akt is phosphorylated by the insulin signaling pathway, it phosphorylates targets such as AKT substrate 160 (AS160) and GSK3β. AMPK is a cellular energy sensor that promotes non-shivering thermogenesis and increases glucose uptake and oxidation (Desjardins and Steinberg, 2018). AKT/AMPK-mediated phosphorylation of AS160 activates translocation of GLUT4 into the plasma membrane, and then GLUT4 uptakes glucose (Eickelschulte et al., 2021). GSK3 is a negative regulator of insulin-mediated glycogen synthesis and glucose homeostasis. The phosphorylation and inactivation of GSK3 can lead to enhanced glycogen synthesis and insulin sensitivity (Rayasam et al., 2009). MMPP enhanced GLUT4 expression and increased glucose uptake in 3T3-L1 MBX cells (Figure 4D and Figure 5). Taken together, these results suggest that MMPP modulates insulin resistance and glucose uptake through the AKT/GSK3/AMPKα signaling pathway (Figure 7).

MMPP treatment promotes adipogenesis in 3T3-L1 MBX cells by modulating adipogenesis-related factors. In addition, MMPP can act as a PPARγ agonist. Side effects of TZDs have drastically reduced the clinical use in controlling type 2 diabetes. Hence, MMPP can contribute to the development of potential new drugs without severe adverse effects against metabolic disorders, including type 2 diabetes.

## Data Availability

The original contributions presented in the study are included in the article/Supplementary Materials, further inquiries can be directed to the corresponding author.

## References

[B1] Achari A. E. Jain S. K. (2017). Adiponectin, a therapeutic target for obesity, diabetes, and endothelial dysfunction. Int. J. Mol. Sci. 18 (6), E1321. 10.3390/ijms18061321 28635626 PMC5486142

[B2] Al Hasan M. Martin P. E. Shu X. H. Patterson S. Bartholomew C. (2021). Type III collagen is required for adipogenesis and actin stress fibre formation in 3T3-L1 preadipocytes. Biomolecules 11 (2), 156. 10.3390/biom11020156 33504048 PMC7911635

[B3] Cariello M. Piccinin E. Moschetta A. (2021). Transcriptional regulation of metabolic pathways via lipid-sensing nuclear receptors PPARs, FXR, and LXR in NASH. Cell. Mol. Gastroenterol. Hepatol. 11 (5), 1519–1539. 10.1016/j.jcmgh.2021.01.012 33545430 PMC8042405

[B4] Chang E. Kim C. Y. (2019). Natural products and obesity: A focus on the regulation of mitotic clonal expansion during adipogenesis. Molecules 24 (6), E1157. 10.3390/molecules24061157 30909556 PMC6471203

[B5] Choi M. K. Kim J. Park H. M. Lim C. M. Pham T. H. Shin H. Y. (2022). The DPA-derivative 11S, 17S-dihydroxy 7, 9, 13, 15, 19 (Z, E, Z, E, Z)-docosapentaenoic acid inhibits IL-6 production by inhibiting ROS production and ERK/NF-κB pathway in keratinocytes HaCaT stimulated with a fine dust PM_10_ Ecotoxicol. Environ. Saf. 232, 113252. 10.1016/j.ecoenv.2022.113252 35104780

[B6] Cox A. J. West N. P. Cripps A. W. (2015). Obesity, inflammation, and the gut microbiota. Lancet. Diabetes Endocrinol. 3 (3), 207–215. 10.1016/S2213-8587(14)70134-2 25066177

[B7] Desjardins E. M. Steinberg G. R. (2018). Emerging role of AMPK in Brown and beige adipose tissue (BAT): Implications for obesity, insulin resistance, and type 2 diabetes. Curr. Diab. Rep. 18 (10), 80. 10.1007/s11892-018-1049-6 30120579

[B8] Eickelschulte S. Hartwig S. Leiser B. Lehr S. Joschko V. Chokkalingam M. (2021). AKT/AMPK-mediated phosphorylation of TBC1D4 disrupts the interaction with insulin-regulated aminopeptidase. J. Biol. Chem. 296, 100637. 10.1016/j.jbc.2021.100637 33872597 PMC8131924

[B9] Farmer S. R. (2005). Regulation of PPARgamma activity during adipogenesis. Int. J. Obes. 29 (1), S13–S16. 10.1038/sj.ijo.0802907 15711576

[B10] Furuhashi M. (2019). Fatty acid-binding protein 4 in cardiovascular and metabolic diseases. J. Atheroscler. Thromb. 26 (3), 216–232. 10.5551/jat.48710 30726793 PMC6402888

[B11] Furuhashi M. Saitoh S. Shimamoto K. Miura T. (2014). Fatty acid-binding protein 4 (FABP4): Pathophysiological insights and potent clinical biomarker of metabolic and cardiovascular diseases. Clin. Med. Insights. Cardiol. 8 (3), 23–33. 10.4137/CMC.S17067 PMC4315049 25674026

[B12] Fyffe S. A. Alphey M. S. Buetow L. Smith T. K. Ferguson M. A. J. Sorensen M. D. (2006). Recombinant human PPAR-beta/delta ligand-binding domain is locked in an activated conformation by endogenous fatty acids. J. Mol. Biol. 356 (4), 1005–1013. 10.1016/j.jmb.2005.12.047 16405912

[B13] Govers R. (2014). Molecular mechanisms of GLUT4 regulation in adipocytes. Diabetes Metab. 40 (6), 400–410. 10.1016/j.diabet.2014.01.005 24656589

[B14] Gunasinghe M. A. Kim A. T. Kim S. M. (2019). Inhibitory effects of vanadium-binding proteins purified from the sea squirt halocynthia roretzi on adipogenesis in 3T3-L1 adipocytes. Appl. Biochem. Biotechnol. 189 (1), 49–64. 10.1007/s12010-019-02982-7 30863985

[B15] Haley M. J. Lawrence C. B. (2016). Obesity and stroke: Can we translate from rodents to patients? J. Cereb. Blood Flow. Metab. 36 (12), 2007–2021. 10.1177/0271678x16670411 27655337 PMC5134197

[B16] Hassan M. Latif N. Yacoub M. (2012). Adipose tissue: Friend or foe? Nat. Rev. Cardiol. 9 (12), 689–702. 10.1038/nrcardio.2012.148 23149834

[B17] Helsen C. Claessens F. (2014). Looking at nuclear receptors from a new angle. Mol. Cell. Endocrinol. 382 (1), 97–106. 10.1016/j.mce.2013.09.009 24055275

[B18] Henke B. R. Blanchard S. G. Brackeen M. F. Brown K. K. Cobb J. E. Collins J. L. (1998). N-(2-Benzoylphenyl)-L-tyrosine PPARgamma agonists. 1. Discovery of a novel series of potent antihyperglycemic and antihyperlipidemic agents. J. Med. Chem. 41 (25), 5020–5036. 10.1021/jm9804127 9836620

[B19] Hishida T. Nishizuka M. Osada S. Imagawa M. (2009). The role of C/EBPdelta in the early stages of adipogenesis. Biochimie 91 (5), 654–657. 10.1016/j.biochi.2009.02.002 19233245

[B20] Huang J. V. Greyson C. R. Schwartz G. G. (2012). PPAR-Gamma as a therapeutic target in cardiovascular disease: Evidence and uncertainty. J. Lipid Res. 53 (9), 1738–1754. 10.1194/jlr.R024505 22685322 PMC3413217

[B21] Kim E. J. Lee D. H. Kim H. J. Lee S. J. Ban J. O. Cho M. C. (2012). Thiacremonone, a sulfur compound isolated from garlic, attenuates lipid accumulation partially mediated via AMPK activation in 3T3-L1 adipocytes. J. Nutr. Biochem. 23 (12), 1552–1558. 10.1016/j.jnutbio.2011.10.008 22405697

[B22] Kores K. Konc J. Bren U. (2021). Mechanistic insights into side effects of troglitazone and rosiglitazone using a novel inverse molecular docking protocol. Pharmaceutics 13 (3), 315. 10.3390/pharmaceutics13030315 33670968 PMC7997210

[B23] Kumar S. K. Rao L. A. Rao B. M. V. LakshmAnA Rao A. (2018). Design, synthesis, biological evaluation and molecular docking studies of novel 3-substituted-5-[(indol-3-yl)methylene]-thiazolidine-2, 4-dione derivatives. Heliyon 4 (9), e00807. 10.1016/j.heliyon.2018.e00807 30258996 PMC6154471

[B24] Lebovitz H. E. (2001). Insulin resistance: Definition and consequences. Exp. Clin. Endocrinol. Diabetes 109 (2), S135–S148. 10.1055/s-2001-18576 11460565

[B25] Lehmann J. M. Moore L. B. Smitholiver T. A. Wilkison W. O. Willson T. M. Kliewer S. A. (1995). An antidiabetic thiazolidinedione is a high-affinity ligand for peroxisome proliferator-activated receptor gamma (Ppar-Gamma). J. Biol. Chem. 270 (22), 12953–12956. 10.1074/jbc.270.22.12953 7768881

[B26] Li T. Zhang L. Jin C. Xiong Y. Cheng Y. Y. Chen K. (2020). Pomegranate flower extract bidirectionally regulates the proliferation, differentiation and apoptosis of 3T3-L1 cells through regulation of PPAR gamma expression mediated by PI3K-AKT signaling pathway. Biomed. Pharmacother. 131, 110769. 10.1016/j.biopha.2020.110769 33152931

[B27] Pettersen E. F. Goddard T. D. Huang C. C. Couch G. S. Greenblatt D. M. Meng E. C. (2004). UCSF Chimera--a visualization system for exploratory research and analysis. J. Comput. Chem. 25 (13), 1605–1612. 10.1002/jcc.20084 15264254

[B28] Poirier P. Giles T. D. Bray G. A. Hong Y. Stern J. S. Pi-Sunyer F. X. (2006). Obesity and cardiovascular disease: Pathophysiology, evaluation, and effect of weight loss: An update of the 1997 American heart association scientific statement on obesity and heart disease from the obesity committee of the council on nutrition, physical activity, and metabolism. Circulation 113 (6), 898–918. 10.1161/CIRCULATIONAHA.106.171016 16380542

[B29] Rayasam G. V. Tulasi V. K. Sodhi R. Davis J. A. Ray A. (2009). Glycogen synthase kinase 3: More than a namesake. Br. J. Pharmacol. 156 (6), 885–898. 10.1111/j.1476-5381.2008.00085.x 19366350 PMC2697722

[B30] Rosen E. D. Sarraf P. Troy A. E. Bradwin G. Moore K. Milstone D. S. (1999). PPAR gamma is required for the differentiation of adipose tissue *in vivo* and *in vitro* . Mol. Cell 4 (4), 611–617. 10.1016/S1097-2765(00)80211-7 10549292

[B31] Rotter V. Nagaev I. Smith U. (2003). Interleukin-6 (IL-6) induces insulin resistance in 3T3-L1 adipocytes and is, like IL-8 and tumor necrosis factor-alpha, overexpressed in human fat cells from insulin-resistant subjects. J. Biol. Chem. 278 (46), 45777–45784. 10.1074/jbc.M301977200 12952969

[B32] Saeedi P. Petersohn I. Salpea P. Malanda B. Karuranga S. Unwin N. (2019). Global and regional diabetes prevalence estimates for 2019 and projections for 2030 and 2045: Results from the international diabetes federation diabetes atlas, 9th edition. Diabetes Res. Clin. Pract. 157, 107843. 10.1016/j.diabres.2019.107843 31518657

[B33] Sanches J. M. Zhao L. N. Salehi A. Wollheim C. B. Kaldis P. (2021). Pathophysiology of type 2 diabetes and the impact of altered metabolic interorgan crosstalk. FEBS J. 10.1111/febs.16306 34847289

[B34] Sanders F. W. B. Griffin J. L. (2016). De novo lipogenesis in the liver in health and disease: More than just a shunting yard for glucose. Biol. Rev. Camb. Philos. Soc. 91 (2), 452–468. 10.1111/brv.12178 25740151 PMC4832395

[B35] Schultze S. M. Hemmings B. A. Niessen M. Tschopp O. (2012). PI3K/AKT, MAPK and AMPK signalling: Protein kinases in glucose homeostasis. Expert Rev. Mol. Med. 14, e1. 10.1017/S1462399411002109 22233681

[B36] Siersbaek R. Nielsen R. Mandrup S. (2012). Transcriptional networks and chromatin remodeling controlling adipogenesis. Trends Endocrinol. Metab. 23 (2), 56–64. 10.1016/j.tem.2011.10.001 22079269

[B37] Son D. J. Kim D. H. Nah S. S. Park M. H. Lee H. P. Han S. B. (2016). Novel synthetic (E)-2-methoxy-4-(3-(4-methoxyphenyl) prop-1-en-1-yl) phenol inhibits arthritis by targeting signal transducer and activator of transcription 3. Sci. Rep. 6, 36852. 10.1038/srep36852 27845373 PMC5109275

[B38] Son D. J. Zheng J. Jung Y. Y. Hwang C. J. Lee H. P. Woo J. R. (2017). MMPP attenuates non-small cell lung cancer growth by inhibiting the STAT3 DNA-binding activity via direct binding to the STAT3 DNA-binding domain. Theranostics 7 (18), 4632–4642. 10.7150/thno.18630 29158850 PMC5695154

[B39] Song Y. S. Lee D. H. Yu J. H. Oh D. K. Hong J. T. Yoon D. Y. (2016). Promotion of adipogenesis by 15-(S)-hydroxyeicosatetraenoic acid. Prostagl. Other Lipid Mediat. 123, 1–8. 10.1016/j.prostaglandins.2016.02.001 26905195

[B40] Trott O. Olson A. J. (2010). AutoDock vina: Improving the speed and accuracy of docking with a new scoring function, efficient optimization, and multithreading. J. Comput. Chem. 31 (2), 455–461. 10.1002/jcc.21334 19499576 PMC3041641

[B41] Unger R. H. Orci L. (2010). Paracrinology of islets and the paracrinopathy of diabetes. Proc. Natl. Acad. Sci. U. S. A. 107 (37), 16009–16012. 10.1073/pnas.1006639107 20798346 PMC2941311

[B42] Wang L. Waltenberger B. Pferschy-Wenzig E. M. Blunder M. Liu X. Malainer C. (2014). Natural product agonists of peroxisome proliferator-activated receptor gamma (PPARγ): A review. Biochem. Pharmacol. 92 (1), 73–89. 10.1016/j.bcp.2014.07.018 25083916 PMC4212005

[B43] Wang S. Dougherty E. J. Danner R. L. (2016). PPARγ signaling and emerging opportunities for improved therapeutics. Pharmacol. Res. 111, 76–85. 10.1016/j.phrs.2016.02.028 27268145 PMC5026568

[B44] Wu Y. Ding Y. Tanaka Y. Zhang W. (2014). Risk factors contributing to type 2 diabetes and recent advances in the treatment and prevention. Int. J. Med. Sci. 11 (11), 1185–1200. 10.7150/ijms.10001 25249787 PMC4166864

[B45] Yadav A. Kataria M. A. Saini V. Yadav A. (2013). Role of leptin and adiponectin in insulin resistance. Clin. Chim. Acta. 417, 80–84. 10.1016/j.cca.2012.12.007 23266767

[B46] Yuan G. Chen X. Li D. (2015). Modulation of peroxisome proliferator-activated receptor gamma (PPAR gamma) by conjugated fatty acid in obesity and inflammatory bowel disease. J. Agric. Food Chem. 63 (7), 1883–1895. 10.1021/jf505050c 25634802

[B47] Zoete V. Grosdidier A. Michielin O. (2007). Peroxisome proliferator-activated receptor structures: Ligand specificity, molecular switch and interactions with regulators. Biochim. Biophys. Acta 1771 (8), 915–925. 10.1016/j.bbalip.2007.01.007 17317294

